# Associations between nutritional deficiencies and food insecurity among adolescent girls: A cross‐sectional study

**DOI:** 10.1002/fsn3.4065

**Published:** 2024-03-07

**Authors:** Mursal Basiry, Pamela J. Surkan, Batoul Ghosn, Ahmad Esmaillzadeh, Leila Azadbakht

**Affiliations:** ^1^ Department of Nutrition Ghazanfar Institute of Health and Science, Ministry of Public Health Kabul Afghanistan; ^2^ Department of Community Nutrition, School of Nutritional Sciences and Dietetics Tehran University of Medical Sciences Tehran Iran; ^3^ Department of International Health Johns Hopkins Bloomberg School of Public Health Baltimore Maryland USA; ^4^ Diabetes Research Center Endocrinology and Metabolism Clinical Sciences Institute, Tehran University of Medical Sciences Tehran Iran

**Keywords:** 24‐h recalls, adolescence, food insecurity, food intake, nutrient deficiency, recommended dietary allowances

## Abstract

There is a research gap in understanding the relationship between nutrient deficiency and food insecurity among adolescent girls in Afghanistan. The objective of this study was to investigate the associations between nutrient deficiencies and food insecurity among middle and high school‐aged girls in Kabul. We conducted a cross‐sectional study of 380 randomly selected 11–18‐year‐old girls attending public schools in grades 6–12. We assessed girls' food insecurity, food and nutrient intake, socioeconomic status, and physical activity. Nutrient consumption was calculated using Nutritionist IV software. Statistical analyses, including one‐way analysis of variance, Chi‐square tests, and t‐tests, were used to assess the association between dietary intake and food insecurity. More than half (52.9%) of the participants were food insecure, with 35.8% experiencing hunger and 17.1% without hunger. Vitamin B3, C, selenium, and iron had the highest sensitivity, specificity, and accuracy and were the best indicators of food insecurity with and without hunger. The most prevalent nutrient deficiencies were vitamin B9 and E, calcium, magnesium, and zinc inadequacies. Food security was positively associated with fruit, vitamins E and K, dairy products (e.g., milk, yogurt, and cheese), meat products (e.g., chicken, meat, red meat, and egg), and nut intake. Our findings suggest that adolescent girls in Kabul may benefit from food programs that enrich nutrients such as B9 and E, calcium, magnesium, and zinc, which were found to be the most prevalent nutrient deficiencies. These findings highlight the importance of addressing food insecurity and nutrient deficiencies among adolescent girls in Afghanistan.

## Graphical abstract

This study aimed to investigate the associations between nutrient deficiencies and food insecurity among middle and high school‐aged girls in Kabul, Afghanistan. Researchers conducted a cross‐sectional study of 380 randomly selected girls attending public schools in grades 6‐12 and found that more than half of the participants were food insecure, with nutrient deficiencies in vitamin B9 and E, calcium, magnesium, and zinc. The study suggests that food programs that enrich these nutrients may benefit adolescent girls in Kabul and highlights the importance of addressing food insecurity and nutrient deficiencies among this population.

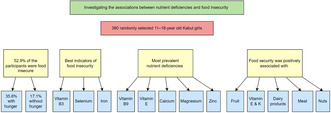

## 1 INTRODUCTION

Food insecurity refers to the inability to acquire enough healthy food to meet one's overall nutritional needs, resulting from insufficient financial or other resources (McGuire et al., 2011). Beyond quantity and quality of food, it also reflects sociocultural and psychological determinants (Food and Agriculture Organization of the United Nations, 2011; Forman et al., 2009; Frongillo & Nanama, 2006; Furness et al., 2004; Liese et al., 2009). Over the last 40 years, the global number of hungry and malnourished people has hovered between 800 million and 1.2 billion people (Food and Agriculture Organization of the United Nations, 2011). In the United States, approximately 1 in 5 adolescents, and over 4 in 10 low‐income adolescents, reside in households facing food insecurity (Dave et al., 2009; Lentz, 2009). This situation poses significant challenges to their growth and development needs, as adequate nutrition is crucial during this critical phase of life. Low‐ and middle‐income countries (LMICs) have the highest rates of malnutrition. Food insecurity and malnutrition affect health and development in both the short‐ and long‐term (Durao et al., 2020). Risk factors for food insecurity in LMICs include poverty, war, governmental policies, environmental degradation, and lack of agricultural development (Food and Agriculture Organization of the United Nations, 2002). According to the 2019 Afghanistan Seasonal Food Security Assessment, 35% of people living in Afghanistan were food insecure (Food Security and Agriculture Cluster, 2019a). In addition, 8.0% of adolescent girls were thin,1.5% were severely thin (Food Security and Agriculture Cluster, 2019a),11.6% were overweight, while only 2.7% were obese (Mashal & Hadad, 2013). Currently, 10.2 million people (33% of the population) have severe acute food insecurity (Food Security and Agriculture Cluster, 2019b). This indicates a considerable increase in food insecurity in Afghanistan from 2013 to 2019.

There are several ways to identify food security. Examples include the United States Department of Agriculture (USDA) questionnaire, the Radimer Kernel questionnaire, the Household Food Security Survey Module (HFSSM), and the Community Childhood Hunger Identification Project (CCHIP) questionnaire (Mashal & Hadad, 2013). These instruments cover different topics such as food accessibility, availability, and sustainability of food security assessments. However, they do not assess the nature of a person's food intake, which may be useful for identifying whether a person is food insecure or not.

Numerous studies have underscored that food insecurity poses a significant nutritional challenge, characterized by a reduced consumption of meat, dairy products, fruits, and vegetables (Aidoo et al., 2013; Food Security and Agriculture Cluster, 2019b; Ramesh et al., 2010) and an increased intake of cost‐effective foods like bread, macaroni, potatoes, legumes, and eggs (Aidoo et al., 2013). Furthermore, the diets of individuals grappling with food insecurity often exhibit deficiencies in vital nutrients, including folic acid, vitamins B12 and C, calcium, iron, magnesium, selenium, and zinc (Craig et al., 2003). These nutritional deficiencies can precipitate a multitude of health issues, thereby establishing a direct correlation between food insecurity and adverse health outcomes. For instance, a comprehensive review exploring the nexus between food insecurity and health outcomes has highlighted that food insecurity is linked to lower nutrient intake, increased risks of certain birth defects, anemia, cognitive impairments, aggression, anxiety, poorer overall health, asthma, behavioral problems, depression, suicidal ideation, and deteriorated oral health in children.

To our knowledge, no research has demonstrated which nutrients in an individual's diet are associated with food insecurity. Given the prevalent issue of food insecurity in Afghanistan, it presents a unique context to investigate its associated nutrient deficiencies. Consequently, our study is focused on discerning the association between these nutritional deficiencies and food insecurity, specifically among female students in Kabul.

## 2 METHODS

Data collection for this cross‐sectional study was carried out from July to December 2019. The purpose was to determine the associations between nutritional deficiencies and food insecurity among adolescent girls in public schools in Kabul, Afghanistan. Kabul is divided into 17 zones based on the cardinal directions. We selected two zones from each cardinal direction, resulting in a total of eight zones (i.e., Zones 2, 3, 9, 10, 11, 12, 13 and 17). We further selected two schools from each zone. A systematic random sampling technique without replacement was used for the selection of zones, schools, and girls. The inclusion criteria were being healthy, a nonimmigrant, and an adolescent female in one of our selected schools. We included girls from grades 6 to 12 who agreed to participate and who did not follow any special diets, such as vegetarian diets (e.g., fasting or weight loss diets) in the past six months. In addition, girls with any potential health problems or history of disease (e.g., hypertension, cardiovascular disease) were excluded. We initially approached 420 students from classes 6 to 12. However, 40 students were excluded prior to data collection. Ten potential participants were excluded due to over‐ and underreporting of total energy intake (either <800 kcal/day or >4200 kcal/day). An additional 30 students were excluded because they refused to participate in the study, did not give consent for study participation, were absent on the date of data collection, returned the USDA form unfilled, or failed to complete their three‐day, 24‐hour dietary intake recall. Therefore, before any data collection and analysis occurred, we had a final sample of 380 girls aged between 11 to 18 years.

This study received ethical approval from the Research Ethics Committee of Tehran University of Medical Sciences, which is overseen by the National Institute for Medical Research Development (Approval number: IR.TUMS.VCR.REC.1398.754). All procedures involving human subjects were conducted in accordance with the guidelines laid down in the Declaration of Helsinki. Written informed consent was obtained from all study participants.

### 2.1 Sample size calculation

The total sample size for this cross‐section study was found to be 373 as per the following formula:

$$n=\frac{{\left(Z_{1-\frac{\alpha }{2}}+Z_{1-\beta }\right)}^{2}\mathrm{pq}\left(r+1\right)}{r{\left(P_{1}-P_{2}\right)}^{2}}=\frac{{\left(1.96+0.84\right)}^{2}0.116\times 0.884\left(1+1\right)}{1{\left(0.116-0.05\right)}^{2}}=373$$

where type one error (*α*) considered as 0.05, *Z*_ (1‐ *α*/2) = 95% = 1.96, *Z*_ (1‐β) = 0.84, *P* = 11.6, *q* = (1–0.116) = 0.884 and *r* = 1. According to National Nutrition Survey (NNS) in (2013) conducted in Afghanistan the Prevalence of overweight 11.6% considered as a base for calculating the sample size in adolescent girls (Aidoo et al., 2013). However, while collecting data from the school we found overall 420 school students gave consent to participate in our study, so we collected data from all of them. In this cross‐sectional study, multistage cluster sampling was performed.

### 2.2 Demographic information

Both fathers' and mothers' education levels were recorded and categorized as illiterate, primary school, secondary school, high school, diploma (high school plus two additional years), bachelor degree (4 years of university), postgraduate degrees (including MA, MS, MSc, MPhil), and PhD. Socioeconomic status (SES) and household income were determined following Ramesh et al.'s method (Ramesh et al., 2010). This method includes information on the number of family members, ownership of modern home appliances, personal car, a house, and number of international trips in the last year. Based on this information, SES was categorized as low, medium, or high. Household income was based on father's occupation. Income was categorized as “unemployed” for fathers without jobs, “low‐income” for fathers who worked as farmers, shopkeepers, metalworkers, repairmen, butchers, bakers, tailors, or in similar professions; “middle income” was used to denote fathers in the government or private sector; and “high income” for fathers who worked as managers, doctors, engineers, experts, lawyers, or judges.

### 2.3 Physical activity assessment

The International Physical Activity Questionnaire (IPAQ) was used to measure adolescent school girls' level of physical activity and was categorized as either very light, light, moderate, or strenuous (Craig et al., 2003). The International Physical Activity questionnaire collects data on physical activity (PA) through an interview. Participants' oral responses are used to calculate their PA in terms of metabolic equivalent hours per week (MET h/wk). The questionnaire covers five domains: job‐related, transportation, housework and maintenance, recreation and leisure time, and time spent sitting. Participants are asked to report on all vigorous and moderate activities as well as time spent sitting during the last 7 days. The IPAQ has shown acceptable validity when assessing levels and patterns of physical activity in adults (Hagströmer et al., 2006).

### 2.4 Anthropometric assessment

Weight, height, waist circumference (WC), and body mass index (BMI) were assessed or calculated for all participants according to standard procedures (Centers for Disease Control and Prevention, 2016). We used calibrated digital scales (SECA 831, Germany) with a precision of 100 g to measure body weight after individuals removed heavy clothing and shoes. To measure height, participants stood against a wall in an upright position without shoes. Waist circumference was measured from the narrowest part of the waist with no pressure applied upon breathing, while participants wore light clothing. We measured BMI using the formula weight in kilograms divided by height in meters squared. Based on the standard World Health Organization (WHO) growth reference curves for school‐aged children and adolescents aged 5–19 years, anthropometric ᴢ‐scores of height‐for‐age (height‐ᴢ) and BMI‐for‐age (BMI‐ᴢ) for girls were calculated (Carlson et al., 1999; Onis et al., 2007). In our study, we utilized the z‐score of BMI‐for‐age to classify the weight status of the participants. The classifications were defined as follows: Obesity: A BMI z‐score greater than 2 standard deviations (SD). Overweight: A BMI z‐score between 1 and 2 SD, normal weight: s BMI z‐score between −2 and 1 SD and underweight: a BMI z‐score less than −2 SD.

### 2.5 Household food security assessment

Household food security over the last year was measured using the 18‐item validated US Department of Agriculture's Food Security Household Health Questionnaire (Carlson et al., 1999). As outlined by Gray Bickle et al., household food security can be categorized as: food secure, food insecure without hunger, food insecure with mild hunger, and food insecure with severe hunger (Bickel et al., 2000). Food insecurity without hunger, food insecurity with mild hunger and food insecurity with severe hunger were classified into an any “food insecurity” group.

This questionnaire evaluates household food security in the last 12 months through patient interviews. This questionnaire in 1995 was introduced as a valid questionnaire for epidemiology studies and this questionnaire has 18 items. The household's food security situation calculated based on the method of “Gray Bickle et al, 2000.” To the answers “often correct” and “sometimes right,” “almost every month,” “some months,” and “yes” positive score (score 1). For answers “not true,” “does not know,” “refuses,” “only one or two months,” and “no” negative score was awarded (score 0). Following the scoring of answers derived from the USDA Household Food Security questionnaire, households divided into four categories; (1) food secure (2) Food insecure without hunger (3) food insecure with mild hunger (4) food insecure with severe hunger (Bickel et al., 2000). The final score of the food security questionnaire is based on the number of positive responses given in Table (McGuire et al., 2011).

We sent the USDA questionnaire and USDA guidance form to each girl's mother. The USDA guidance form guides the respondent in the filing of the form. If the mother is illiterate, other educated members of her family, for example, her father or siblings can help fill out the USDA questionnaire. The USDA questionnaire includes three sections. The first section is related to food status and includes 3 questions. The second section is about adulthood, which is divided into two parts of 5 questions and 2 questions. The final third section is about a child younger than 18 years and is further divided into two parts of 3 and 5 questions. If the respondent fills in the first part and even gives a positive answer to a single question, they are eligible to fill in the second adult part. Otherwise, move directly to fill in the last third section of the questionnaire. If you fill in three sections of the questionnaire, the overall positive number of the USDA questionnaire will be 18. Finally, according to the number of positive responses, calculate the food security score, and then divide the household food security status into four categories based on the following individual scores: (McGuire et al., 2011) food secure, (Liese et al., 2009) food insecure without hunger, (Forman et al., 2009) food insecure with mild hunger, and (Furness et al., 2004) food insecure with severe hunger.

### 2.6 Food and dietary intakes

We assessed the dietary habits of the adolescent schoolgirls by administering the 24‐h Food and Dietary Recall Questionnaire over three separate days. This approach allowed us to capture a more comprehensive picture of their typical food and dietary intakes. The dietary intake of the schoolgirls was assessed through a series of interviews, during which each interviewer utilized a kit containing various serving sizes to measure food. This kit included items such as a can, a palm‐sized piece of bread, a tablespoon, a teaspoon, a dipper spoon, a plate, a bowl, a glass, images of food, household food items, a meter, and stationery.

Before each interview, we familiarized the participants with the serving sizes. After the initial interview, the completed questionnaires were reviewed, and any errors or omissions were addressed in subsequent meetings with the same participant. The quantity of each food item was converted into grams using a household scale and then analyzed using the Nutritionist 4 (NUT4) software. In the final analysis, we excluded 10 girls who had reported total energy intake outside the acceptable range (either less than 800 or more than 4200 kcal/day) (Saraf‐Bank et al., 2017).

In this study, we categorized food items into several groups and subgroups. The bread/grain group was divided into seven subgroups: whole wheat bread, whole‐grain bread, biscuit, rice, macron, whole‐grain cereals, and refined flour. The fruit category was split into two subgroups: one encompassing all fruits and the other including all fruits along with juices. The vegetable group was further divided into seven subgroups: potato and other starchy vegetables, eggplant, tomato, cucumber, beans, yellow vegetables, green leafy vegetables, and other vegetables.

The meats and meat substitutes were classified into four subgroups: red and organ meat, chicken, fish, and egg. Lastly, the “dairy” group was divided into three subgroups: all types of milk, yogurt, and cheese (Tavakoli et al., 2016).

Recommended Dietary Allowances (RDAs) are the levels of intake of essential nutrients that, based on scientific knowledge, are judged by the Food and Nutrition Board to be adequate to meet the known nutrient needs of practically all healthy persons (National Research Council (US) Subcommittee on the Tenth Edition of the Recommended Dietary Allowances, 1989). In this study at first means of 3 days nutrients intake were calculated for each nutrient, then according to the nutrient RDA cutoff points suggested for adolescent girls, we calculated the 75% and 50% of RDA (shown in supplementary file) for each individual. Finally, the frequency of subjects in entire food insecure level and food insecure with and without hunger in the established cutoff points of nutrient deficiency was determined by cross‐tabulation. According to this, sensitivity, specificity, and accuracy were calculated as per the following formula:

1. **Sensitivity = a/a + c × 100**
  Sensitivity = a (true positive) / a+c (true positive + false negative)
  Sensitivity = Probability of being test positive when food insecurity present.
2. **Specificity = d/b + d × 100**
  Specificity = d (true negative) / b+d (true negative + false positive)
  Specificity = Probability of being test negative when food insecurity absent.
3. **Accuracy = a + d/a + b + c + d × 100**
  Accuracy = is how close you are to the true value

**Table:** 

	Food Insecure	Food Secure	Total
Test positive	True positive (a)	False positive (b)	Total test positive (a + b)
Test negative	False negative (c)	True negative (d)	Total test negative (c + d)
Total	Total food insecure (a + c)	Total food secure (b + d)	Total population (a + b + c + d)

The sensitivity of each nutrient deficiency was defined as the proportion of the total number of subjects with given food insecurity who were identified correctly by the same nutrient deficiency. The specificity of each nutrient deficiency was defined as the proportion of the total subjects without food insecurity who were correctly identified by the same nutrient deficiency. Accuracy of each nutrient deficiency was defined as the proportion of the total subjects with and without the food insecurity who were correctly identified by the same nutrient deficiency (shown in supplementary file).

### 2.7 Recommended daily allowance

The United States National Institutes of Health (https://ods.od.nih.gov/index.aspx) and the Institutes of Medicine have established Recommended Daily Allowances (RDA) for various nutrients according to gender and age. We calculated the 75% RDA limit for each vitamin and mineral, as shown in Table 1.

**TABLE 1:** Recommended daily allowance (75%) of vitamins and minerals for females.

Age	9–13 years	14–18 years
Vitamins		
Vitamin A (RAE/d)	450	525
Vitamin B1 (mg/d)	0.675	0.75
Vitamin B2 (mg/d)	0.675	0.75
Vitamin B3 (mg/d)	9	10.5
Vitamin B6 (mg/d)	0.75	0.9
Folate or B9 (mcg)	225	300
Vitamin B12 (μg/d)	1.35	1.8
Vitamin C (mg/d)	33.75	48.75
Vitamin E (mg/d)	8.25	11.25
Vitamin K(mcg/d)	45	56.25
Minerals		
Calcium (mg/d)	975	975
Iron (mg/d)	6	11.25
Magnesium (mg/d)	180	270
Potassium (mg/d)	0.003	0.003
Selenium (mcg/d)	0.03	0.04
Zinc (mg/d)	6	6.75

Abbreviation: RAE, retinol activity equivalents.

### 2.8 Vitamins and minerals

We determined sensitivity, specificity, and accuracy for each vitamin and mineral (shown in supplementary file). The sensitivity, specificity, and accuracy for each vitamin and mineral were identified for the following groups, that is, any food insecurity, food insecure with hunger and food insecure without hunger. We used cross‐tabulations to examine the frequency of adolescent girls with and without all types of food insecurity, food insecurity with hunger or food insecurity without hunger based on established cut points for vitamin and minerals according to the formula shown in Table 6. In this study, the sensitivity, specificity, and accuracy of each vitamin or mineral were calculated as the proportions of correctly identified adolescent girls in various categories of food insecurity.

### 2.9 Statistical analysis

Excel 2013 software was used for data entry. Nutritionist IV software was used to obtain the amount of nutrients consumed by each person. SPSS 26 (SPSS Inc) software used for the analysis of data. The normal distribution of variables was evaluated by using the histogram and Kolmogorov–Smirnov test. Chi‐square was used to determine the general characteristics of the individuals per food security levels. A chi‐square test was used to obtain a comparison of the distribution of individuals among the multivariate in case of qualitative variables. One‐way ANOVA was used to determine nutrients deficiency of the subjects compared among the various food insecurity levels. After the One‐way ANOVA test, the Tukey HSD test used to compare groups with each other. Finally, sensitivity, specificity, and accuracy for nutrient deficiency was calculated to predict food insecurity and find which nutrients have the highest percentage of deficiency in food insecure levels. Nutritionist IV software was used to calculate nutrients consumed by each person. One‐way analysis of variance, Chi‐square tests, and t‐tests were used for statistical analyses conducted in SPSS version 16 (SPSS Inc.). Data were reported as mean ± standard deviation for continuous variables and as frequency (percentages) for categorical variables.

## 3 RESULTS

### 3.1 Household food security status

Less than 50% of adolescent schoolgirls had food security (*n* = 179; 47.1%). A considerable number were food insecure (*n* = 201; 52.9%) without hunger (*n* = 136; 35.8%), food insecure with mild hunger (*n* = 45; 11.8%), and food insecure with severe hunger (*n* = 20; 5.3%) (Table 2 and supplementary file).

**TABLE 2:** Frequency of food security status among adolescent schoolgirls.

Food security status^a^	Frequency (%) (*n* = 380)
A. Food secure	179 (47.1)
B. Food insecure	201 (52.9)

^a^ USDA Food Security Household Health Questionnaire based on the methods of Gray Bickle et al.

### 3.2 Household characteristics

Levels of food insecurity were significantly associated with household income (*p* = .001) and SES (*p* = .0001) as shown in Table 3. Although the largest number of food‐secure households was categorized as having medium SES (*n* = 129; 51.6%), food insecurity was also observed among middle‐income households (*n* = 82; 52.6%), In households experiencing food insecurity, fathers with an education level of secondary school or lower were more prevalent compared to educated fathers in food‐secure households (*p* = .011) Similarly, food insecurity was more prevalent in households with uneducated mothers, suggesting that higher maternal education improved food security (*p* = .014). In addition, grade in school was also related to food security level (*p* = .029). Engaging in very light to moderate physical activity was related to higher household food security (*p* = .031). However, household size and geographic zone were not related to food security levels.

**TABLE 3:** Households characteristics among food secure and insecure groups.

Variables	School girls (*n* = 380) Mean	Food secure vs. food insecure		*p*‐value (chi‐square)
		Food secure *n* (%)	Food insecure *n* (%)	
Age (years)	14.86	15.07 ± 0.15	14.64 ± 0.13	**.035**
Household size				
<7 person	7.632	92 (45.8)	109 (54.2)	.581
>7 person		87 (48.6)	92 (51.4)	
Grade in school				
6	9.06	22 (46.8)	25 (53.2)	**.029**
7		26 (44.1)	33 (55.9)	
8		19 (33.3)	38 (66.7)	
9		18 (38.3)	29 (61.7)	
10		24 (45.3)	29 (54.7)	
11		36 (57.1)	27 (42.9)	
12		34 (63.0)	20 (37)	
Zone				
2	0.158	14 (40.0)	21 (60)	.161
3		18 (31.6)	39 (68.4)	
9		18 (52.9)	16 (47.1)	
10		29 (46.8)	33 (53.2)	
11		29 (53.8)	25 (46.3)	
12		16 (42.1)	22 (57.9)	
13		29 (54.7)	24 (45.3)	
17		26 (55.3)	21 (44.7)	
Father's education level				
Illiterate	4.83	10 (27.0)	27 (73)	**.011**
Primary school		5 (29.4)	12 (70.6)	
Secondary school		2 (33.3)	4 (66.7)	
High school (12y)		40 (42.6)	54 (57.4)	
Diploma (14 y)		24 (50.0)	24 (50)	
Bachelor (16 y)		63 (50.4)	62 (49.6)	
M.Sc.		30 (68.2)	14 (31.8)	
PhD		5 (55.6)	4 (44.4)	
Mother's education level				
Illiterate	3.39	36 (34.0)	70 (66)	**.014**
Primary school		21 (47.7)	23 (52.3)	
Secondary school		5 (45.5)	6 (54.5)	
High school (12 y)		62 (54.4)	52 (45.6)	
Diploma (14 y)		12 (36.4)	21 (63.6)	
Bachelor (16 y)		36 (57.1)	27 (42.9)	
M.Sc.		6(75.0)	2 (25)	
PhD		1(100)	0 (0)	
Socioeconomic Score				
Low	0.86	20 (22.2)	70 (77.8)	**.0001**
Medium		129 (51.6)	121 (48.4)	
High		30 (75)	10 (25)	
Income				
Unemployed	1.96	5 (31.3)	11 (68.8)	**.001**
Low income		32 (33.7)	63 (66.3)	
Medium income		74 (47.4)	82 (52.6)	
High income		68 (60.2)	45 (39.8)	
Physical activity				
Very light	1.50	36 (38.3)	58 (61.7)	**.031**
Light		50 (52.1)	46 (47.9)	
Moderate		39 (41.1)	56 (58.9)	
Strenuous		54 (56.8)	41 (43.2)	

*Note*: *p* < .05 indicates a significant value.

### 3.3 Food and dietary intakes

We found that there were direct associations between food secure and insecure groups with dairy, fruit, and meat intake groups (*p* < .0001) as shown in Table 4. Individuals who were food insecure had lower food intake from dairy, fruits, and meat food groups compared to food‐secure individuals. In addition, we also found that being food secure (or insecure) was directly associated with intake of food items such as egg, milk, nuts, red meat, and yogurt (*p* < .0001). Cheese and poultry consumption were positively related to food security and insecurity (*p* = .001 and *p* = .024, respectively). Households that consumed milk, yogurt, and cheese (83.32 gr ± 5.17 gr), fruits or juices (83.46 gr ± 6.22 gr) and red meat, chicken, fish, and eggs (21.17 gr ± 1.64 gr) were more food secure relative to food insecure households that consumed fewer of these foods.

**TABLE 4:** Food and dietary intake among food secure and insecure groups.

Food and dietary intake	School girls (*n* = 380) (mean ± SE)	Food secure vs. food insecure		*p*‐value (*t*‐test)
		Food secure (mean ± SE)	Food insecure (mean ± SE)	
Macronutrient				
Carbohydrate (g/d)	245.6 ± 4.18	250.2 ± 76.2	241.5 ± 85.9	.297
Vitamins				
Vitamin A (RAE/d)	390.3 ± 26.0	401.0 ± 41.7	380.7 ± 32.46	.697
Vitamin B1 (mg/d)	1.407 ± 0.02	1.441 ± 0.04	1.373 ± 0.03	.270
Vitamin B2 (mg/d)	0.857 ± 0.23	0.877 ± 0.03	0.840 ± 0.03	.424
Vitamin B3 (mg/d)	13.67 ± 0.30	14.02 ± 0.47	13.35 ± 0.37	.270
Vitamin B6 (mg/d)	0.768 ± 0.35	0.785 ± 0.05	0.752 ± 0.04	.651
Vitamin B9 (folate) (mcg)	139.7 ± 5.63	140.0 ± 7.99	139.4 ± 7.93	.960
Vitamin B12 (μg/d)	4.355 ± 0.18	4.469 ± 0.23	4.254 ± 0.27	.603
Vitamin C (mg/d)	65.81 ± 7.54	69.81 ± 12.2	62.25 ± 9.17	.617
Vitamin E (mg/d)	2.131 ± 0.29	2.831 ± 0.53	1.514 ± 0.30	**.028**
Vitamin K (mcg/d)	34.85 ± 2.03	31.13 ± 2.42	38.14 ± 3.17	.086
Minerals				
Calcium (mg/d)	509.0 ± 12.6	532.4 ± 22.3	488.0 ± 12.9	.079
Iron (mg/d)	10.03 ± 0.22	10.26 ± 0.35	9.823 ± 0.28	.322
Magnesium (mg/d)	136.6 ± 5.02	144.6 ± 7.92	129.5 ± 6.34	.135
Potassium (mg/d)	1.633 ± 0.95	1.732 ± 2.01	1.545 ± 1.70	.326
Selenium (mcg/d)	0.063 ± 0.00	0.027 ± 0.00	0.025 ± 0.00	.739
Zinc (mg/d)	3.971 ± 0.84	4.035 ± 0.11	3.915 ± 0.12	.480
Food groups				
Bread & grains group				
White bread, whole wheat bread, biscuits, macron, rice & refined flour	168.0 ± 2.92	167.3 ± 4.25	168.6 ± 4.03	.836
Dairy Group				
Milk, yogurt & cheese	51.86 ± 3.13	83.32 ± 5.17	23.71 ± 2.32	**<.0001**
Fruit group				
Fruit & fruit juices	49.62 ± 3.78	83.46 ± 6.22	19.33 ± 3.26	**<.0001**
Meat Group				
Red meat, chicken, fish & egg	13.50 ± 0.92	21.17 ± 1.64	6.652 ± 0.631	**<.0001**
Vegetable group				
Beans, cucumber, eggplant, green leafy vegetables, potato, pumpkin, tomato, & yellow vegetables	59.28 ± 1.12	59.32 ± 1.70	59.24 ± 1.49	.969
Individual food items				
Beans (g/d)	51.28 ± 2.03	50.67 ± 2.99	51.82 ± 2.78	.779
Cheese (g/d)	2.092 ± 0.26	3.044 ± 0.47	1.243 ± 0.26	**.001**
Chicken (g/d)	8.418 ± 1.12	11.11 ± 1.85	6.019 ± 1.33	**.024**
Egg (g/d)	37.47 ± 2.97	59.12 ± 5.32	18.19 ± 2.29	**<.0001**
Green leafy vegetables (g/d)	6.820 ± 0.55	7.275 ± 0.79	6.414 ± 0.76	.436
Milk (g/d)	40.31 ± 3.08	66.51 ± 5.27	17.11 ± 2.51	**<.0001**
Nuts (Almonds, cashew, peanut, pistachios, walnut etc.) (g/d)	0.422 ± 0.07	0.798 ± 0.14	0.087 ± 0.03	**<.0001**
Potato (g/d)	40.33 ± 2.06	41.24 ± 3.09	39.51 ± 2.76	.675
Red meat (g/d)	7.355 ± 0.97	12.90 ± 1.88	2.412 ± 0.58	**<.0001**
Rice (g/d)	112.4 ± 4.13	113.9 ± 6.06	111.0 ± 5.66	.725
White bread (g/d)	188.7 ± 3.74	186.6 ± 5.32	190.6 ± 5.27	.591
Yellow vegetables (g/d)	1.789 ± 0.23	2.049 ± 0.37	1.558 ± 0.30	.304
Yogurt (g/d)	26.55 ± 2.42	42.14 ± 4.23	12.60 ± 2.19	**<.0001**

Abbreviation: RAE, retinol activity equivalents.

*Note*: *p* < .05 indicates a significant value.

### 3.4 Recommended daily allowance

Percentages of adolescent girls, who did not meet the RDA limits of 75% and 50% among food secure, food insecure without hunger and food insecure with hunger groups varied (shown in supplementary file). All adolescent girls reached both the 50% and 75% of the RDA for carbohydrates (i.e., all types of bread, rice, spaghetti, and sugar). For vitamin B1, all adolescent girls met the 50% RDA cutoff, but none met the 75% RDA cutoff.

The percentage of adolescent girls who did not meet the 75% and 50% RDA cutoffs was significant for many vitamins and minerals. Among adolescent girls, 15.5% did not reach the RDA cutoffs of 75% (*p* = .007) and 50% (*p* = .025) for vitamin B2. Also, 15.5% did not meet the RDA limit of 75% (*p* = .002) for vitamin B12.

### 3.5 Predictors of food insecurity

A comparison of sensitivity, specificity, and accuracy for vitamins and minerals according to the 50% and 75% RDA cutoffs was performed (shown in supplementary file). Using the 75% RDA cutoffs, only vitamins B2, B3, C and iron had high enough sensitivity, specificity, and accuracy to predict food insecurity, food insecurity with hunger or food insecurity without hunger. However, using the 50% RDA cutoffs, only selenium had high enough sensitivity, specificity, and accuracy to predict either any food insecurity, food insecurity with hunger or food insecurity without hunger.

### 3.6 Risk of food insecurity

The risk of adolescent girls who had food insecurity, food insecurity with hunger and food insecurity without hunger levels not meeting 75% of the RDA are shown in Table 5 and supplementary file. We found a 2.3 higher odds of vitamin B2 deficiency among adolescent girls with food insecurity with hunger not meeting 75% of RDA (OR = 2.29; 95% CI: 1.33, 3.96). However, among participants who did not meet 75% of the RDA cutoff, risk of “food insecurity” and “food insecurity without hunger” did not increase. We did not observe a significant increase in total food insecurity and food insecurity without hunger levels among those who did not meet 75% of the RDA. Girls with any food insecurity (with or without hunger) had 1.53 times the odds of not meeting 75% of RDA for vitamin B3 (OR = 1.53; 95% CI: 1.01, 2.3). The associations between vitamin B3 and food insecurity with hunger and food insecurity without hunger were not significant. Food insecure girls had twofold higher odds of not meeting 75% of the RDA for vitamin B12 (OR = 2.06; 95% CI: 1.14, 3.72). Similarly, for girls with food insecurity with hunger had 2.81 higher odds of not meeting 75% of the RDA for vitamin B12 (OR = 2.81; 95%CI: 1.5, 5.28). Girls with food insecurity had 4.37 higher odds of not meeting 75% of RDA for vitamin E (OR = 4.37; 95%CI: 1.2, 15.9). We did not observe a significant association between vitamin E and food insecurity with hunger or with food insecurity without hunger. There were no other significant increases in the odds for food insecurity levels by inadequate intake of other nutrients (Vitamins A, B1, B6, B9, C, K) and minerals (magnesium, iron, selenium and zinc).

**TABLE 5:** Odds ratios for food insecurity by inadequate intake of different nutrients.

Variables	Any food insecurity	
	≥RDA	Not meeting 75% of the RDA
	OR (95% CI)	OR (95% CI)
Vitamins		
Vitamin A (RAE/d)	1 (1)	0.74 (0.42, 1.29)
Vitamin B1 (mg/d)	1 (1)	1.78 (0.16, 19.8)
Vitamin B2 (mg/d)	1 (1)	1.32 (0.87, 2.01)
Vitamin B3 (mg/d)	1 (1)	1.53 (1.01, 2.3)
Vitamin B6 (mg/d)	1 (1)	1.24 (0.72, 2.10)
Vitamin B9 (folate) (mcg)	1 (1)	0.99 (0.37, 2.64)
Vitamin B12 (μg/d)	1 (1)	2.06 (1.14, 3.72)
Vitamin C (mg/d)	1 (1)	0.87 (0.58, 1.31)
Vitamin E (mg/d)	1 (1)	4.37 (1.2, 15.9)
Vitamin K (mcg/d)	1 (1)	1.02 (0.62, 1.68)
Minerals		
Magnesium (mg/d)	1 (1)	2.91 (0.59, 1.32)
Iron (mg/d)	1 (1)	0.88 (0.59, 1.32)
Selenium (mcg/d)	1 (1)	0.64 (0.37, 1.11)
Zinc (mg/d)	1 (1)	0.94 (0.41, 9.46)

Abbreviation: RAE, retinol activity equivalents.

### 3.7 Nutrient deficiency

The proportion of individuals identified with inadequate nutrient intakes at different levels of food insecurity are shown in supplementary file. Based on 50% and 75% of the RDA, the highest proportion of individuals with any food insecurity and food insecurity with and without hunger corresponded to the inadequacy of vitamin B9 and E, calcium, magnesium, and zinc.

## 4 DISCUSSION

Our study provides a comprehensive analysis of food insecurity and its association with nutrient deficiencies among our participants. More than half (52.9%) of the participants were found to be food insecure, with a significant proportion experiencing hunger (35.8%) and others without hunger (17.1%). We identified vitamins B3 and C, selenium, and iron as the most reliable indicators of food insecurity, exhibiting the highest sensitivity, specificity, and accuracy.

The most prevalent nutrient deficiencies observed were those of vitamin B9 and E, calcium, magnesium, and zinc. Notably, our study found that 29.9% and 7.4% of adolescent girls have anemia and folate (vitamin B9) deficiency, respectively. We also found a positive association between food security and the intake of fruits, vitamins E and K, dairy products (such as milk, yogurt, and cheese), meat products (including chicken, meat, red meat, egg), and nuts. These findings underscore the complex interplay between dietary habits, nutrient intake, and food security.

Our study found that 29.9% and 7.4% of adolescent's girls have anemia and folate (vitamin B9) deficiency, respectively, which is classified by the WHO as a moderate public health problem (Afghanistan Ministry of Public Health U, 2013). According to Afghanistan's National Nutrition Survey, 40% of women of reproductive age suffer from anemia (Mashal & Hadad, 2013). Likewise, the WHO has highlighted that anemia is a severe public health problem in women of reproductive age in Afghanistan (Anwary et al., 2021; Mashal & Hadad, 2013). Although adolescent girls consumed vegetables in this study, their average intake was only 59.28 grams a day. This is far below the 3–5 servings (each serving = 160 g) recommended from different vegetable groups in a 24‐h period (Haghighatdoost et al., 2013). In fact, folate deficiency in this study may be due to insufficient folate‐rich intake of vegetables. Our findings align with other studies conducted globally. For instance, a study conducted in Kathmandu found that the overall prevalence of anemia among adolescent girls was 17.4%, with a higher prevalence in schools where Weekly Iron Folic Acid Supplementation (WIFAS) was not implemented (Khanal et al., 2024). This suggests that interventions like WIFAS can play a significant role in reducing anemia and folate deficiency. In Indonesia, a multisectoral project implementing weekly iron supplementation for adolescent girls in West Java showed promising results in reducing anemia (Roche et al., 2018). This project highlighted the importance of school‐based interventions, given that adolescents rarely access preventive health services, but a large percentage are enrolled in secondary school. These studies underscore the importance of public health interventions and the consumption of folate‐rich foods in combating anemia and folate deficiency among adolescent girls.

We found vitamin E deficiency to be most prevalent among participants with any food insecurity. Furthermore, we observed a significant correlation between food insecurity and both vitamin E and nut consumption. Because vitamin E is synthesized by plants and obtained through dietary sources such as nuts, spinach, whole grains, olive oil, and sunflower oil (Keen & Hassan, 2016), these results suggest that less dietary intake of nuts might lead to food insecurity and vice versa. Similarly, the association we found between household income and food security levels also suggests an incapacity for households in Kabul to purchase foods containing vitamin E. In comparison with the existing literature, a study (Beyene, 2023) also highlighted the adverse effects of food insecurity on health outcomes, emphasizing the importance of food security and nutrition in improving health outcomes. Similarly, a systematic review (Lopes et al., 2023) concluded that food insecurity is associated with micronutrient deficiency, which aligns with our findings on vitamin E deficiency.

Globally, calcium and magnesium deficiency lead to common health problems (Sachs, 2001; White & Broadley, 2009; World Health Organization, 2002, 2004). These minerals are important for normal bone and tooth development, and insufficient dietary intake can lead to osteomalacia and osteoporosis (Nieves, 2005). The long‐term deficiency of these minerals may promote a variety of chronic diseases (Allgrove, 2004; Nieves, 2005; Pittas et al., 2007). The most common dietary sources of calcium and magnesium are dairy products, various meats, such as chicken, turkey, pork, and beef, eggs, and whole grains (Whitton et al., 2011). Among minerals, we found the highest prevalence of low calcium and magnesium in girls with food insecurity. We found that the percentage of individuals whose magnesium did not reach >75% of the RDA was also related to food security level. Our study found significant associations between consumption of milk, yogurt, egg, red meat, chicken, and cheeses with both food security and insecurity levels. These findings suggest that less dietary intake of milk, yogurt, egg, red meat, chicken, cheeses, and whole grain are responsible for calcium and magnesium deficiency among school‐aged girls, which may result in the bone and dental diseases if not controlled (Castiglioni et al., 2013; Rude & Olerich, 1996). In comparison with the existing literature, a study by Keri Marshall et al. (Marshall et al., 2020) also highlighted the adverse effects of inadequate calcium and vitamin D intake on the risk of osteoporosis among older Americans living in poverty with food insecurities. Similarly, a review (Castiglioni et al., 2013) emphasized that magnesium deficiency contributes to osteoporosis by directly affecting crystal formation and bone cells, and indirectly impacting the secretion and activity of parathyroid hormone and promoting low‐grade inflammation.

Zinc, required by the body in trace amounts, is necessary for the activity of countless enzymes and for the maintenance of normal metabolism and physical growth. In this study, we found high levels of zinc deficiency were associated with all food insecurity classifications. This may be due to low consumption of or poor absorption of zinc in the diets of girls attending public schools. In our study, we found food insecure girls had higher levels of vegetable consumption, and lower intake of meat products than food secure girls. This suggests that a possible reason for the poor zinc absorption in our study may be due to a fiber‐rich diet or low consumption of meat products. A previous study among Afghan women reported 23.4% prevalence of zinc deficiency (Levitt et al., 2010). According to a national survey, 15.1% of Afghan women of reproductive age have zinc deficiency (Afghanistan Ministry of Public Health U, 2013). Similarly, many published studies have observed vitamin and mineral inadequacy (i.e., vitamin B9, calcium, magnesium, and zinc) to be associated with food insecurity (Jamalikandazi et al., 2016; Kirkpatrick & Tarasuk, 2008; M'Kaibi et al., 2015). In comparison with the existing literature, a study (Beyene, 2023) highlighted the adverse effects of food insecurity on health outcomes, emphasizing the importance of food security and nutrition in improving health outcomes. Similarly, a review (Knez & Stangoulis, 2023) presented an overview of the magnitude of zinc deficiency with a particular emphasis on present global challenges, current recommendations for zinc intake, and factors that affect dietary requirements. These comparisons underscore the critical role of diet and income in determining food security levels and nutritional status, particularly in relation to zinc intake. Further research is needed to explore these associations in greater depth and develop interventions to address these nutritional deficiencies.

We found vitamin K was associated with food security, as well as with food insecurity. Vegetables and fruits are good sources of vitamin K and its various forms. Green leafy vegetables supply 40% to 50% of total intake of phylloquinone. Other sources of vitamin K are vegetables oils such as soybean, cottonseed, canola, and olive oil. Although menaquinone is common in dairy products (Lipsky, 1994; Schurgers et al., 1999), nearly 50% of vitamin K in the body is supplied by intestinal bacteria, suggesting the importance of this bacteria for normal vitamin K supply. Vitamin K deficiency may be responsible for various chronic diseases (Lipsky, 1994; Silver, 1988). This aligns with the findings of a study (Mladěnka et al., 2022) which discussed the physiological role of vitamin K, its deficiency, and its impact on health. Similarly, a review (Bruno, 2016) highlighted the prevalence of vitamin K deficiency and insufficiency, and recommended increased intake. These comparisons underscore the critical role of diet in determining food security levels and nutritional status, particularly in relation to vitamin K intake. Further research is needed to explore these associations in greater depth and develop interventions to address these nutritional deficiencies.

Many vitamins are destroyed at higher cooking temperatures (Levitt et al., 2010). In contrast, vitamin K remains stable regardless of cooking temperatures, but is degraded by exposure to light (Marsh, 1933). In prior studies, low intakes of fruits and leafy vegetables have been observed among food insecure households (Dharod et al., 2011). However, in our study, we did not find green leafy vegetable intake to be associated with food insecurity levels. Hence, given this fact, it appears that vitamin K deficiency in school‐age girls might be attributable to preexisting unhealthy gut bacteria. Further research is needed to understand the role of gut bacteria in the production of vitamin K among school‐aged children.

In our study, we found that not reaching >75% of the RDA for vitamin B2 (or riboflavin), B3 (niacin), and B12 (cobalamin) were related to food insecurity. The primary dietary sources of riboflavin are foods of animal origin, and to a lesser extent, green leafy vegetables (Bellows et al., 2012; D'Souza & Jolliffe, 2013). In Afghanistan, one major obstacle is the price of these types of foods. Dairy products, animal‐based foods, and seafood are very expensive, and for this reason, low‐income households are unable to consume these types of foods (Bellows et al., 2012; D'Souza & Jolliffe, 2013). Prior research has also shown lower intake of fruits, dairy and meat products to be associated with household food insecurity (Dharod et al., 2011, 2013; Nnakwe & Yegammia, 2002).

In our study >50% of the participants showed mineral deficiencies, including iron. We did not observe iron to be associated with food security or insecurity levels. Globally, iron deficiency is the most common nutrient deficiency. Another study reported that 48.4% of Afghan women were iron‐deficient (Levitt et al., 2010). This could be due to low consumption of zinc‐ and iron‐containing foods (Levitt et al., 2010). In general, zinc and iron deficiencies tend to occur together (Levitt et al., 2010). Dark green leafy vegetables, meats, and whole grains are good sources of iron. In addition to consumption of iron‐deficient foods, poor dietary absorption of iron is another reason for iron deficiency (Lai et al., 2012). Prior research has documented associations between iron and zinc deficiency with food insecurity among adolescents (Eicher‐Miller et al., 2009; Kirkpatrick & Tarasuk, 2008). However, our study did not show an association between iron and zinc with food insecurity levels. This might be partially explained by the consumption of poor iron and zinc‐containing foods or poor absorption due to a fiber‐rich diet.

In our study, <50% of participants were food secure (*n* = 179; 47.1%). At the same time, a considerable proportion were found to be food insecure (*n* = 201; 52.9%). These results were similar to a previous study, indicating that half of the population of Afghanistan lives below the poverty line, leading systematically to high food insecurity (WFP, 2020). Regional conflict and community insecurity have been observed as main causes of food insecurity (WFP, 2020). In this study, we also observed significant differences between the living standards of rural and urban cities (WFP, 2020). Nearly 12.5 million people in Afghanistan were found to be food insecure (WFP, 2020). Similar results have been observed in recent research on Afghan immigrants in Iran; over 60% suffered from moderate to severe food insecurity, 37% were moderately food insecure, and around 23% were food secure (Omidvar et al., 2013). Another report by the World Bank highlighted the rising food insecurity in 2023, with domestic food price inflation remaining high (World Bank, 2024). This could potentially explain the high rates of food insecurity as high food prices can limit access to nutritious food, leading to deficiencies like the vitamin E deficiency observed in this study.

In our sample, a greater proportion of mothers were illiterate (*n* = 116 (27.9%)) than fathers (*n* = 37 (9.7%)). Both fathers' and mothers' education levels were associated with food insecurity (*p* = .01 for both). In Afghanistan, large gender gaps in access, educational achievement, and continuing education exist, at the expense of girls (United Nations Educational, Scientific and Cultural Organization, 2016). Additional obstacles to completing or exercising one's education may be related to a variety of factors such as war and insecurity, poverty, geographical isolation, minority status, disability, early marriage and pregnancy, gender‐based violence, and traditional approaches to the status and role of women (Oskorouchi et al., 2018; Parvazian et al., 2017; Ross et al., 2012; United Nations Educational, Scientific and Cultural Organization, 2016; van Hek et al., 2016). A recent study in Afghanistan showed that 49% of men and 15% of women were literate, pointing to similar social determinants such as war, insecurity, gender‐based violence, poverty, and traditional beliefs (Zulfacar, 2006; Central Statistics Organization (CSO), Ministry of Public Health, 2015). Another study emphasized the influence of parental perceptions of the food environment on food decisions among disadvantaged families (Ravikumar et al., 2022), suggesting that parental education could influence family food decisions and consequently, food security. Moreover, a study on household food insecurity showed that it not only affects the normal physical growth of young children but also adversely affects their intellectual capacity and social skills (Tamiru & Belachew, 2017). This underscores the importance of addressing food insecurity, which is associated with parents' education levels as per your findings.

Despite collecting data from different geographical zones in Kabul, we were unable to find an association between the zone and food security levels. This might be due to an insufficient number of zones under study or to the fact that Kabul, being capital of Afghanistan, has relatively good access to food resources across the city.

We found household income and socioeconomic status to be significantly associated with food security levels (p = .001 and p = .0001, respectively). Approximately half of Afghanistan's population lives in poverty, which is a major factor affecting food security (WFP, 2020). Similar results were observed in a recent study among Afghan immigrants in Iran, where a significant association was found between socioeconomic status, income, and household food insecurity (Omidvar et al., 2013). Notably, several studies show high rates of food insecurity associated with household income and education level (Amaza et al., 2006; Dastgiri et al., 2006; Gibson, 2003; Pankomera et al., 2009; Webb et al., 2008).

In our study, household size was not associated with food insecurity. Contradictory results have been reported regarding the relationship between food insecurity and household size. For example, one study showed a negative relationship between food insecurity and household size in Nigeria, suggesting that households with larger family sizes were more food secure than those with smaller household sizes (Amaza et al., 2006). Other studies have reported negative associations between household size and food security, possibly explained by increased food requirements for a larger number of people (Aidoo et al., 2013; Ajao et al., 2010; Chaput et al., 2007; Pankomera et al., 2009; Tabrizi et al., 2018).

This study is the first to determine which nutrient deficiencies are most related to food insecurity. Further, it is the first study of its kind in Kabul Afghanistan to examine the relationship between food insecurity and nutrient deficiency among adolescent girls. The current study has several limitations that should be considered. First, this study was cross‐sectional, which does not allow for causal inference. In future studies, the possible effects of unknown confounders should be considered. Additionally, our study was conducted with only girls, so may not be generalizable to boys. selection bias may exist as we sampled girls only from public schools. Data were not collected from private schools because they tend to include more children with higher SES. Therefore, it is likely that there may be different predictors of food security in this group. Additionally, there is a possibility of recall bias as we used 24‐h dietary recall which depends on memory.

Finally, our research highlights the need for interventions to address food insecurity and improve access to nutritious foods for adolescent girls. This could involve the implementation of programs to provide food assistance, education on healthy eating, and support for local food systems. Additionally, policies aimed at reducing poverty and improving economic opportunities for families could help to address the root causes of food insecurity. By addressing these issues, we can help to ensure that adolescent girls have access to the nutritious foods they need to support their growth and development.

## 5 CONCLUSION

In this cross‐sectional study, we identified specific nutrient deficiencies related to food insecurity. However, more research is needed to understand these associations in more detail, including to understand potential sex differences. Longitudinal research is also needed in to provide stronger evidence with respect to these associations.

## AUTHOR CONTRIBUTIONS

**Mursal Basiry:** Data curation (equal); formal analysis (equal); investigation (equal); writing – original draft (lead). **Pamela Surkan:** Supervision (equal); writing – review and editing (equal). **Batoul Ghosn:** Writing – review and editing (equal). **ahmad esmaillzadeh:** Supervision (equal); writing – review and editing (equal). **Leila Azadbakht:** Conceptualization (lead); formal analysis (equal); methodology (lead); project administration (lead); supervision (lead); writing – review and editing (equal).

## FUNDING INFORMATION

NA.

## CONFLICT OF INTEREST STATEMENT

All authors declare that they have no conflict of interest.

## ETHICS STATEMENT AND CONSENT TO PARTICIPATE

This study was conducted according to the guidelines laid down in the Declaration of Helsinki and all procedures involving research study participants were approved by the Research Council and Ethical Committee of the School of Nutrition and Food Science, Tehran University of Medical Sciences, Tehran, Iran. Written informed consent was attained from all subjects/patients.

## Supporting information


Data S1.

## Data Availability

The data that support the findings of this study are available on request from the corresponding author. The data are not publicly available due to privacy or ethical restrictions.
