# Supplementary material for: Associations between nutritional deficiencies and food insecurity among adolescent girls: A cross‐sectional study
Source: Food Sci Nutr. 2024 Mar 7;12(7):4623–36. doi: 10.1002/fsn3.4065 (PMC11266893; doi:10.1002/fsn3.4065)
Supplement: Supplementary file 1 — Data S1. [file FSN3-12-4623-s001.docx]

| **Table 1: Recommended Daily Allowance of vitamins and minerals for females** | | | | |
| --- | --- | --- | --- | --- |
| **Age** | **9 to 13 years** | | **14 to 18 years** | |
| **RDA** | **100%** | **50%** | **100%** | **50%** |
| **Vitamins** |  |  |  |  |
| Vitamin A (RAE/d) | 600 | 300 | 700 | 350 |
| Vitamin B1 (mg/d) | 0.9 | 0.45 | 1.0 | 0.5 |
| Vitamin B2 (mg/d) | 0.9 | 0.45 | 1.0 | 0.5 |
| Vitamin B3 (mg/d) | 12 | 6 | 14 | 7 |
| Vitamin B6 (mg/d) | 1.0 | 0.5 | 1.2 | 0.6 |
| Folate or B9 (mcg) | 300 | 150 | 400 | 200 |
| Vitamin B12 (µg/d) | 1.8 | 0.9 | 2.4 | 1.2 |
| Vitamin C (mg/d) | 45 | 22.5 | 65 | 32.5 |
| Vitamin E (mg/day) | 11 | 5.5 | 15 | 7.5 |
| Vitamin K(mcg/day) | 60 | 30 | 75 | 37.5 |
| **Minerals** |  |  |  |  |
| Calcium (mg/d) | 1300 | 650 | 1300 | 650 |
| Iron (mg/d) | 8 | 4 | 15 | 7.5 |
| Magnesium (mg/d) | 240 | 120 | 360 | 180 |
| Potassium (mg/d) | 0.0045 | 0.002 | 0.0047 | 0.002 |
| Selenium (mcg/day) | 0.04 | 0.02 | 0.055 | 0.02 |
| Zinc (mg/d) | 8 | 4 | 9 | 4.5 |

| **Table 2: Sensitivity and specificity** | | | |
| --- | --- | --- | --- |
|  | **Any food insecurity*** | **Food security** | **Total** |
| **Positive test** | True positive (a) | False positive (b) | Total test positive (a + b) |
| **Negative test** | False negative (c) | True negative (d) | Total test negative (c + d) |
| **Total** | Total any food insecurity* (a + c) | Total food secure (b + d) | Total adolescent girls  (a + b + c + d) |
|  | **Sensitivity (a / a + c)** | **Specificity**  **(d / b + d)** | **Accuracy**  **(a + d / a + d + b + c)** |
| * This includes food insecure with hunger and food insecure without hunger | | | |

| **Table 3: Frequency of food security status among adolescent schoolgirls.** | |
| --- | --- |
| **Food security status*** | **Frequency (%)**  **(n = 380)** |
| A. Food secure | 179 (47.1) |
| B. Food insecure without hunger | 136 (35.8) |
| C. Food insecure with mild hunger | 45 (11.8) |
| D. Food insecure with severe hunger | 20 (5.3) |
| * USDA Food Security Household Health Questionnaire based on the methods of Gray Bickle et al. | |

| **Table 4: Proportion of adolescent girls who didn’t meet the 75% and 50% RDA cutoffs for vitamins and minerals** | | | | | | | | | | |  |
| --- | --- | --- | --- | --- | --- | --- | --- | --- | --- | --- | --- |
| **Variables** | **Girls who didn’t meet 75% RDA limit** | | | | | **Girls who didn’t meet 50% RDA limit** | | | | | |
|  | **School girls***  **n (%)** | **Food Security Status** | | | **P-value**  **(Chi-square)** | **School girls***  **n (%)** | **Food Security Status** | | | **P-value**  **(Chi-square)** | |
|  |  | **Food secure**  **n (%)** | **Food insecure without hunger**  **n (%)** | **Food insecure with hunger**  **n (%)** |  |  | **Food secure**  **n (%)** | **Food insecure without hunger**  **n (%)** | **Food insecure with hunger**  **n (%)** |  |  |
| **Vitamins** | | | | | | | | | | |  |
| Vitamin A (RAE/d) | 319 (83.8) | 154 (40.5) | 109 (28.6) | 56 (14.7) | 0.321 | 232 (60.9) | 114 (30) | 74 (19.4) | 44 (11.5) | 0.119 | |
| Vitamin B1 (mg/d) | 3 (0.6) | 1 (0.2) | 1 (0.2) | 1 (0.2) | 0.744 | - | - | - | - | - | |
| Vitamin B2 (mg/d) | 149 (39) | 64 (16.8) | 49 (12.8) | 36 (9.4) | **0.007** | 38 (10) | 12 (3.1) | 14 (3.6) | 12 (3.1) | **0.025** | |
| Vitamin B3 (mg/d) | 174 (45.6) | 72 (18.9) | 69 (18.1) | 33 (8.6) | 0.121 | 6 (1.4) | 1 (0.2) | 2 (0.5) | 3 (0.7) | 0.079 | |
| Vitamin B6 (mg/d) | 314 (82.5) | 145 (38.1) | 111 (29.2) | 58 (15.2) | 0.219 | 120 (31.4) | 58 (15.2) | 41 (10.7) | 21 (5.5) | 0.904 | |
| Vitamin B9 (folate) (mcg) | 363 (95.5) | 171 (45) | 130 (34.2) | 62 (16.3) | 0.998 | 319 (83.9) | 152 (40) | 114 (30) | 53 (13.9) | 0.816 | |
| Vitamin B12 (µg/d) | 59 (15.5) | 19 (5) | 21 (5.5) | 19 (5) | **0.002** | 27 (6.9) | 11 (2.8) | 10 (2.6) | 6 (1.5) | 0.717 | |
| Vitamin C (mg/d) | 210 (55.1) | 102 (26.8) | 69 (18.1) | 39 (10.2) | 0.381 | 107 (28) | 53 (13.9) | 33 (8.6) | 21 (5.5) | 0.415 | |
| Vitamin E (mg/day) | 364 (95.6) | 166 (43.6) | 134 (35.2) | 64 (16.8) | 0.053 | 363 (95.5) | 166 (43.6) | 133 (35) | 64 (16.8) | 0.108 | |
| Vitamin K(mcg/day) | 299 (78.6) | 140 (36.8) | 105 (27.6) | 54 (14.2) | 0.631 | 241 (63.3) | 118 (31) | 83 (21.8) | 40 (10.5) | 0.687 | |
| **Minerals** | | | | | | | | | | |  |
| Calcium (mg/d) | 374 (98.3) | 175 (46) | 134 (35.2) | 65 (17.1) | 0.461 | 323 (85) | 149 (39.2) | 119 (31.3) | 55 (14.4) | 0.574 | |
| Iron (mg/d) | 202 (53) | 98 (25.7) | 70 (18.4) | 34 (8.9) | 0.837 | 54 (14) | 22 (5.7) | 21 (5.5) | 11 (2.8) | 0.567 | |
| Magnesium (mg/d) | 366 (96.2) | 169(44.4) | 133 (35) | 64 (16.8) | 0.173 | 311 (81.6) | 144 (37.8) | 112 (29.4) | 55 (14.4) | 0.743 | |
| Selenium (mcg/day) | 317 (83.3) | 155 (40.7) | 111 (29.2) | 51 (13.4) | 0.249 | 229 (60.1) | 112 (29.4) | 83 (21.8) | 34 (8.9) | 0.474 | |
| Zinc (mg/d) | 356 (93.6) | 168 (44.2) | 127 (33.4) | 61 (16) | 0.984 | 259 (68) | 118 (31) | 97 (25.5) | 44 (11.5) | 0.592 | |
| * Total number of adolescent girls who did not meet the 75% or 50% RDA limit for each vitamin or mineral; (n = 380). | | | | | | | | | | |  |

| **Table 5: Sensitivity, specificity and accuracy of vitamins and minerals in determining food insecurity** | | | | | | |
| --- | --- | --- | --- | --- | --- | --- |
| **Variables** | **75% RDA** | | | **50 % RDA** | | |
|  | **Sensitivity** | **Specificity** | **Accuracy** | **Sensitivity** | **Specificity** | **Accuracy** |
| **Food insecurity (both with and without hunger)** | | | | | | |
| **Vitamins** | | | | | | |
| Vitamin A (RAE/d) | 82 | 13.9 | 50 | 58.7 | 36.3 | 52.3 |
| Vitamin B1 (mg/d) | 0.99 | 99.4 | 47.3 | 0 | 100 | 47.1 |
| Vitamin B2 (mg/d) | **42.7** | **64.2** | **52.8** | 12.9 | 93.2 | 50.2 |
| Vitamin B3 (mg/d) | **50.7** | **59.7** | **55** | 2.4 | 99.4 | 48.1 |
| Vitamin B6 (mg/d) | 83.5 | 18.9 | 53.1 | 30.8 | 67.5 | 48.1 |
| Vitamin B9 (folate) (mcg) | 95.5 | 4.4 | 52.6 | 83 | 15 | 51 |
| Vitamin B12 (µg/d) | 19 | 89.3 | 52.6 | 7.9 | 92.7 | 47.8 |
| Vitamin C (mg/d) | **53.7** | **43** | **48.6** | 26.8 | 70.3 | 47.3 |
| Vitamin E (mg/day) | 98.5 | 7.2 | 95.7 | 98 | 7.2 | 55.2 |
| Vitamin K (mcg/day) | 79.1 | 21.7 | 51 | 61.1 | 34 | 48.4 |
| **Minerals** | | | | | | |
| Calcium (mg/d) | 99 | 2.2 | 53.4 | 86.5 | 16.7 | 53.6 |
| Iron (mg/d) | **51.7** | **45.2** | **53.1** | 15.9 | 87.7 | 49.7 |
| Magnesium (mg/d) | 98 | 5.5 | 54.4 | 83 | 19.5 | 53.1 |
| Potassium (mg/d) | 0 | 100 | 47.1 | 0 | 100 | 47.1 |
| Selenium (mcg/day) | 80.5 | 13.4 | 48.9 | **58.7** | **57** | **48.1** |
| Zinc (mg/d) | 93.5 | 6.1 | 52.3 | 70.1 | 34 | 53 |
| **Food insecurity with hunger** | | | | | | |
| **Vitamins** | | | | | | |
| Vitamin A (RAE/d) | 86.1 | 13.9 | 21.3 | 67.6 | 36.3 | 28.6 |
| Vitamin B1 (mg/d) | 1.53 | 99.4 | 47.1 | 0 | 100 | 47.1 |
| Vitamin B2 (mg/d) | **55.3** | **64.2** | **39.7** | 18.4 | 93.2 | 47.1 |
| Vitamin B3 (mg/d) | **50.7** | **59.7** | **36.8** | 4.6 | 99.4 | 47.1 |
| Vitamin B6 (mg/d) | 89.2 | 18.9 | 24.2 | 32.3 | 67.5 | 37.3 |
| Vitamin B9 (folate) (mcg) | 95.3 | 4.4 | 18.4 | 81.5 | 15 | 21 |
| Vitamin B12 (µg/d) | 29.2 | 89.3 | 47.1 | 9.2 | 92.7 | 45.2 |
| Vitamin C (mg/d) | **60** | **43** | **30.5** | 32.3 | 70.3 | 38.6 |
| Vitamin E (mg/day) | 98.4 | 7.2 | 20.2 | 98.4 | 7.2 | 20.2 |
| Vitamin K(mcg/day) | 83 | 21.7 | 24.4 | 61.5 | 34 | 26.5 |
| **Minerals** |  |  |  |  |  |  |
| Calcium (mg/d) | 100 | 2.2 | 18.1 | 84.6 | 16.7 | 22.3 |
| Iron (mg/d) | **52.3** | **45.2** | **30.2** | 16.9 | 87.7 | 44.2 |
| Magnesium (mg/d) | 98.1 | 5.5 | 10.1 | 84.6 | 19.5 | 23.6 |
| Potassium (mg/d) | 100 | 50 | 47.1 | 0 | 100 | 47.1 |
| Selenium (mcg/day) | 78.4 | 13.4 | 19.7 | **52.3** | **35.7** | **25.7** |
| Zinc (mg/d) | 93.8 | 6.1 | 45.2 | 67.6 | 34 | 27.6 |
| **Food insecurity without hunger** | | | | | | |
| **Vitamins** | | | | | | |
| Vitamin A (RAE/d) | 80.1 | 13.9 | 35.2 | 54.4 | 36.3 | 36.5 |
| Vitamin B1 (mg/d) | 0.73 | 99.4 | 47.1 | 0 | 100 | 47.1 |
| Vitamin B2 (mg/d) | **36** | **64.2** | **43.1** | 10.2 | 93.2 | 47.6 |
| Vitamin B3 (mg/d) | **50.7** | **59.7** | **46.3** | 1.4 | 99.4 | 47.1 |
| Vitamin B6 (mg/d) | 81.6 | 18.9 | 38.1 | 30.1 | 67.5 | 42.6 |
| Vitamin B9 (folate) (mcg) | 95.5 | 4.4 | 36.3 | 83.8 | 15 | 37.1 |
| Vitamin B12 (µg/d) | 15.4 | 89.3 | 47.6 | 7.3 | 92.7 | 46.3 |
| Vitamin C (mg/d) | **50.7** | **43** | **38.4** | 24.2 | 70.3 | 41.8 |
| Vitamin E (mg/day) | 98.5 | 7.2 | 38.6 | 97.7 | 7.2 | 38.4 |
| Vitamin K (mcg/day) | 77.2 | 21.7 | 37.8 | 61 | 34 | 37.8 |
| **Minerals** |  |  |  |  |  |  |
| Calcium (mg/d) | 98.5 | 2.2 | 36.3 | 87.5 | 16.7 | 39.2 |
| Iron (mg/d) | **51.4** | **45.2** | **39.7** | 15.4 | 87.7 | 46.8 |
| Magnesium (mg/d) | 97.7 | 5.5 | 37.6 | 82.3 | 19.5 | 38.6 |
| Potassium (mg/d) | 0 | 100 | 47.1 | 0 | 100 | 47.1 |
| Selenium (mcg/day) | 81.6 | 13.4 | 35.5 | **61.7** | **55.6** | **38.9** |
| Zinc (mg/d) | 93.3 | 6.1 | 36.3 | 71.3 | 34 | 41.5 |

| **Table 6: Odds ratios for food insecurity by inadequate intake of different nutrients** | | | | |
| --- | --- | --- | --- | --- |
| **Variables** | **Food insecurity with hunger** | | **Food insecurity without hunger** | |
|  | **≥ RDA** | **Not meeting**  **75 % of the RDA** | **≥ RDA** | **Not meeting**  **75 % of the RDA** |
| **Vitamins** | **OR (95%CI)** | **OR (95%CI)** | **OR (95%CI)** | **OR**  **(95%CI)** |
| Vitamin A (RAE/d) | 1 (1) | 1.23 (0.57, 2.64) | 1 (1) | 0.65 (0.37, 1.13) |
| Vitamin B1 (mg/d) | 1 (1) | 2.44 (0.21, 27.3) | 1 (1) | 1.11 (0.1, 12.4) |
| Vitamin B2 (mg/d) | 1 (1) | 2.29 (1.33, 3.96) | 1 (1) | 0.8 (0.52, 1.24) |
| Vitamin B3 (mg/d) | 1 (1) | 1.27 (0.74, 2.17) | 1 (1) | 1.36 (0.89, 2.07) |
| Vitamin B6 (mg/d) | 1 (1) | 1.88 (0.81, 4.34) | 1 (1) | 0.9 (0.52, 1.56) |
| Vitamin B9 (folate) (mcg) | 1 (1) | 0.96 (0.26, 3.44) | 1 (1) | 1.02 (0.37, 2.83) |
| Vitamin B12 (µg/d) | 1 (1) | 2.81 (1.5, 5.28) | 1 (1) | 0.89 (0.54, 1.75) |
| Vitamin C (mg/d) | 1 (1) | 1.26 (0.73, 2.17) | 1 (1) | 0.75 (0.49, 1.14) |
| Vitamin E (mg/day) | 1 (1) | 2.77 (0.35, 21.5) | 1 (1) | 3.49 (0.77, 15.8) |
| Vitamin K (mcg/day) | 1 (1) | 1.38 (0.68, 2.78) | 1 (1) | 0.85 (0.51, 1.42) |
| **Minerals** | | | | |
| Magnesium (mg/d) | 1 (1) | 2.75 (0.35, 21.4) | 1 (1) | 2.09 (0.57, 7.63) |
| Iron (mg/d) | 1 (1) | 0.96 (0.56, 1.63) | 1 (1) | 0.9 (0.59, 1.37) |
| Selenium (mcg/day) | 1 (1) | 0.67 (0.34, 1.3) | 1 (1) | 0.81 (0.52, 1.24) |
| Zinc (mg/d) | 1 (1) | 1.03 (0.34, 3.13) | 1 (1) | 0.92 (0.39, 2.17) |

| **Table 7: Percentage of individuals with food insecurity who had** **inadequate nutrient intake** | | | | | | |
| --- | --- | --- | --- | --- | --- | --- |
| **Variables** | **75% RDA** | | | **50 % RDA** | | |
|  | **Food insecure** | **FI with hunger** | **FI without hunger** | **Food insecure** | **FI with hunger** | **FI without hunger** |
| **Vitamins** | | | | | | |
| Vitamin A (RAE/d) | 43.4 | 14.7 | 28.6 | 31 | 11.5 | 19.4 |
| Vitamin B1 (mg/d) | 0.5 | 0.26 | 0.26 | 0 | 0 | 0 |
| Vitamin B2 (mg/d) | 22.6 | 9.4 | 12.8 | 6.8 | 3.1 | 3.6 |
| Vitamin B3 (mg/d) | 26.8 | 8.6 | 18.1 | 1.2 | 0.7 | 0.5 |
| Vitamin B6 (mg/d) | 44.2 | 15.2 | 29.2 | 16.3 | 5.5 | 10.7 |
| Folate or B9 (mcg) | 50.5 | 16.3 | 34.2 | 43.9 | 13.9 | 30 |
| Vitamin B12 (µg/d) | 10.5 | 5 | 5.5 | 4.1 | 1.5 | 2.6 |
| Vitamin C (mg/d) | 28.4 | 10.2 | 18.1 | 14.2 | 5.5 | 8.6 |
| Vitamin E (mg/day) | 52.1 | 16.8 | 35.2 | 51.8 | 16.8 | 35 |
| Vitamin K (mcg/day) | 41.8 | 14.2 | 27.6 | 32.3 | 10.5 | 21.8 |
| **Minerals** | | | | | | |
| Calcium (mg/d) | 52.3 | 17.1 | 35.2 | 45.7 | 14.4 | 31.3 |
| Iron (mg/d) | 27.3 | 8.9 | 18.4 | 8.3 | 2.8 | 5.5 |
| Magnesium (mg/d) | 51.8 | 16.8 | 35 | 43.9 | 14.4 | 29.4 |
| Potassium (mg/d) | 0 | 0 | 0 | 0 | 0 | 0 |
| Selenium (mcg/day) | 42.6 | 13.4 | 29.2 | 31 | 8.9 | 22.1 |
| Zinc (mg/d) | 49.4 | 16.0 | 33.4 | 37 | 11.5 | 25.5 |
